# Cancer immunotherapy insights: key takeaways from the ADSCC bone marrow and cellular therapy congress 2024

**DOI:** 10.3389/fimmu.2025.1650023

**Published:** 2026-01-20

**Authors:** Gisela Maria Suárez Formigo, Zaima Mazorra, Cinthia Olexen, Tamara Menéndez, Loubna Abdel Hadi, Rupert Handgretinger, Antonio Alfonso Bencomo Hernandez, Inas El Najjar, George Coukos, Helen Sabzevari, Catherine Bollard, Daniel Couriel, David Wald, Mohamad Hamieh, Javier Briones, Fatima Al Kaabi, Yendry Ventura-Carmenate

**Affiliations:** 1 Abu Dhabi Stem Cells Center, Abu Dhabi, United Arab Emirates; 2 College of Medicine and Health Sciences, United Arab Emirates University, Al Ain, United Arab Emirates; 3 Oncology/Oncology, Children’s University Hospital, Tübingen, Germany; 4 St. Jude Children’s Research Hospital, Memphis, TN, United States; 5 Ludwig Institute for Cancer Research, Lausanne University Hospital, Lausanne, Switzerland; 6 Department of Oncology, Lausanne University Hospital, Lausanne, Switzerland; 7 Precigen, Inc., Germantown, MD, United States; 8 Children’s National Hospital, Washington, DC, United States; 9 George Washington University, Washington, DC, United States; 10 Translational Research and Innovation, GW Cancer Center, Washington, DC, United States; 11 Huntsman Cancer Institute, University of Utah, Salt Lake, UT, United States; 12 Kure.ai, Cleveland, OH, United States; 13 Immune Oncology Program, Case Comprehensive Cancer Center, Cleveland, OH, United States; 14 Department of Pediatrics, Meyer Cancer Center, Weill Cornell Medicine, New York, NY, United States; 15 Hospital de la Santa Creu i Sant Pau, Josep Carreras Leukemia Research Institute, Barcelona, Spain; 16 Hematology Service, Hospital de la Santa Creu i Sant Pau, Barcelona, Spain

**Keywords:** adoptive cell transfer, cancer immunotherapy, CAR-T cells, precision medicine, Tumor-infiltrating Lymphocytes (TILs), Tumor microenvironment (TME)

## Abstract

The rising global cancer burden underscores the urgent need for innovative and effective therapies. Molecular and cellular immunology advances have revolutionized cancer immunotherapy, transforming laboratory discoveries into clinical breakthroughs. The Second Bone Marrow Transplant and Cellular Therapy Congress, held in Abu Dhabi, United Arab Emirates (UAE), on October 26^th^ - 27^th^, 2024, and sponsored by the Abu Dhabi Stem Cells Center (ADSCC), convened global experts to discuss cutting-edge developments in adoptive cell transfer (ACT); chimeric antigen receptor T-cell (CAR-T) therapy, tumor-infiltrating lymphocyte (TIL) engineering and T-cell receptor (TCR) innovations. Discussions covered key challenges such as tumor microenvironment (TME) resistance, antigen escape, manufacturing complexity, cost-effectiveness, and accessibility. Experts emphasized the crucial role of biomarker identification in optimizing patient selection and improving treatment efficacy. Additionally, emerging strategies were highlighted to enhance the durability and specificity of cellular therapies, including next-generation CAR-T designs, combination approaches, and novel gene-editing technologies. With over 2,300 participants from academia, research, and healthcare, the event fostered international collaborations and knowledge exchange. The ADSCC continues to play a pivotal role in integrating advanced cellular therapies into healthcare systems, contributing to the expansion of precision oncology in the UAE and beyond. This review analyzes the latest advances in immunotherapy, highlighting their clinical impact, challenges, and future directions in the evolving landscape of cancer treatment, as debated during the congress.

## 1 Introduction

The global burden of oncological diseases continues to rise at an alarming rate. As reported by the World Health Organization, an estimated 20 million new cancer cases and 9.7 million cancer-related deaths were recorded in 2022 (1). According to the latest estimates available from the Global Cancer Observatory, lung cancer was the most common cancer worldwide, with 2.5 million new cases, representing 12.4% of all new cases. Female breast cancer ranked second (2.3 million cases; 11.6%), followed by colorectal cancer (1.9 million cases; 9.6%), prostate cancer (1.5 million cases; 7.3%), and stomach cancer (970,000 cases; 4.9%) (2).

In the United Arab Emirates (UAE), breast cancer remained one of the leading causes of cancer-related deaths, accounting for approximately 11.6% of annual cancer fatalities in 2019 (3). A study published in 2024 reported that 1,139 cases of breast cancer were recorded in the UAE in 2021, representing 20.3% of all malignant cases diagnosed that year (4). Additionally, colorectal cancer was the second most common cause of cancer-related mortality in both sexes (3). Alarmingly, evidence suggests a rising cancer incidence among younger adults in the UAE, with an increasing prevalence of breast and colorectal cancer in this demographic (5, 6). Among hematological malignancies, leukemia and non-Hodgkin lymphoma are also among the top 10 most common cancers in the country (7). Recognizing the urgency of these trends, the UAE Government has launched nationwide initiatives to promote early cancer detection through its health authorities. In parallel, the government has actively supported the development and accessibility of innovative therapeutic strategies, ensuring that patients receive advanced treatments and experience improved clinical outcomes. Together, these efforts reflect a comprehensive and forward-looking national approach to oncology, integrating prevention, early diagnosis, and access to advanced therapies to combat the growing cancer burden.

In response to this global shift toward cutting-edge cancer therapies, advanced immunotherapy-based cell therapies have emerged as transformative treatment options, harnessing the body’s natural defenses to target and eliminate malignant cells. Among these breakthroughs, adoptive cell transfer (ACT) has demonstrated remarkable efficacy, offering new hope for patients with limited treatment options (8). ACT encompasses a spectrum of strategies, including chimeric antigen receptor (CAR) T-cell therapy, tumor-infiltrating lymphocytes (TILs), and engineered T-cell receptor (TCR) therapies, which collectively represent a paradigm shift in cancer treatment. This progress aligns with the UAE’s commitment to integrating next-generation oncology treatments into its healthcare system, reinforcing its role in the advancement of cancer immunotherapy.

Reflecting on these advancements, the ADSCC Congress 2024 convened leading experts to discuss the latest breakthroughs in CAR-T therapy, tumor-infiltrating lymphocytes (TILs), and next-generation T-cell engineering. As cancer immunotherapy continues to evolve, these emerging treatments are reshaping the oncological landscape, providing novel therapeutic avenues. The congress provided a comprehensive overview of these innovations while addressing key challenges, including tumor microenvironment (TME) resistance, antigen escape, and therapy accessibility. Additionally, discussions focused on optimizing treatment protocols, developing cost-effective solutions, and broadening patient eligibility for cellular therapies.

Beyond summarizing congress proceedings, this review integrates mechanistic, clinical, and translational perspectives to contextualize how next-generation adoptive cell therapies are reshaping cancer treatment paradigms, with particular emphasis on scalability, accessibility, and real-world implementation.

## 2 Advances in adoptive cell transfer

### 2.1 Foundations of ACT, CAR-T, TIL and TCR

ACT has emerged as a transformative immunotherapy approach, leveraging the patient’s immune system to target and eliminate cancer cells. This strategy primarily involves isolation, genetic modification (if necessary), and *ex vivo* expansion of autologous T cells before reinfusing them into the patient. ACT has demonstrated sustained clinical success, including the regression of large, vascularized metastatic tumors, highlighting its transformative potential in cancer therapy (8). Within the realm of ACT, chimeric antigen receptor; CAR-T cell therapy has emerged as a landmark advance, combining synthetic receptor engineering with autologous T-cell infusion to redirect immune specificity toward predefined tumor-associated surface antigens. CAR-T therapy is primarily used for the treatment of leukemia, lymphoma, and multiple myeloma (9–11), where clinical trials and post-approval real-world studies have demonstrated efficacy in heavily pretreated patients. Specifically, overall response rates range from approximately 80–85%, with complete response rates of approximately 45–55%, depending on the disease indication and CAR construct, as reported in clinical studies of CD19- and BCMA-directed CAR-T therapies in B-cell acute lymphoblastic leukemia, large B-cell lymphoma, and multiple myeloma (9–11). These data are derived from clinical phase I–II trials and subsequent global multicenter studies, underscoring the remarkable clinical success of CAR-T therapy in hematologic malignancies and fueling extensive research into its potential application in solid tumors.

However, the efficacy of antigen-targeted therapies in solid tumors faces significant challenges, primarily due to the paucity of targets for CAR-T therapy, the antigenic heterogeneity and the immunosuppressive TME. These factors limit the therapeutic impact and remain major obstacles to the broader application of CAR-T in solid malignancies. In response to these challenges, TIL therapy has emerged as a promising alternative to overcome these limitations. This approach involves isolating TILs directly from the tumor tissue and then expanding them *ex vivo* in the presence of cytokines such as interleukin-2 (IL-2) to promote their proliferation. Before TIL infusion, patients undergo lymphodepletion chemotherapy to enhance the therapy’s efficacy. Unlike single antigen-targeting therapies, TILs can recognize multiple undefined tumor antigens, eliciting a robust, nearly clonal or oligoclonal T-cell response. This greater flexibility and adaptability allow TILs to effectively identify and destroy heterogeneous tumor populations, offering new hope for overcoming tumor immune evasion and antigenic variability. Despite the promise of TIL therapy, challenges remain in inefficiently isolating and expanding tumor-specific T cells for all patients, prompting the search for alternative strategies to enhance adoptive cell therapy (12, 13).

One such promising approach is the modified T-cell receptor (TCR) therapy, which provides a viable solution when natural anti-tumor T cells cannot be activated or expanded in sufficient numbers. Unlike TIL therapy, which relies on isolating and expanding naturally occurring tumor-reactive T cells, TCR therapy involves genetically modifying patient-derived T cells to express a new T-cell receptor specifically designed to recognize and target cancer-associated antigens. By equipping T cells with this enhanced antigen-recognition capability, TCR therapy offers a more precise and personalized treatment strategy, potentially overcoming the barriers associated with insufficient T-cell activation. This innovative approach expands the therapeutic landscape of ACT, providing renewed hope for patients with tumors that may otherwise evade immune detection (13).

While TIL, CAR-T, and TCR therapies represent promising advancements in ACT, challenges such as tumor immune evasion, complex manufacturing processes, and high treatment costs remain significant hurdles to their widespread implementation (13, 14). Beyond these limitations, additional factors further constrain their clinical translation, including treatment-related toxicities that can impact clinical management and accessibility. The most common and clinically relevant adverse events are cytokine release syndrome (CRS) and immune effector cell–associated neurotoxicity syndrome (ICANS). These immune-mediated toxicities may require hospitalization, intensive care support, and targeted interventions such as interleukin-6 (IL-6) receptor blockade with monoclonal antibodies, including tocilizumab. CRS and ICANS have been extensively characterized in pivotal phase I–II clinical trials and post-approval studies of CD19- and BCMA-directed CAR-T therapies, forming the basis for current toxicity grading and management guidelines (15). Nevertheless, despite these challenges and limitations, the therapeutic responses observed in patients, even in advanced stages of cancer, underline the tremendous potential of these therapies and justify continued exploration and innovation. To harness their benefits, efforts should focus on overcoming tumor escape mechanisms, streamlining manufacturing processes, and reducing costs to enhance accessibility (14, 16).

**Figure 1:**
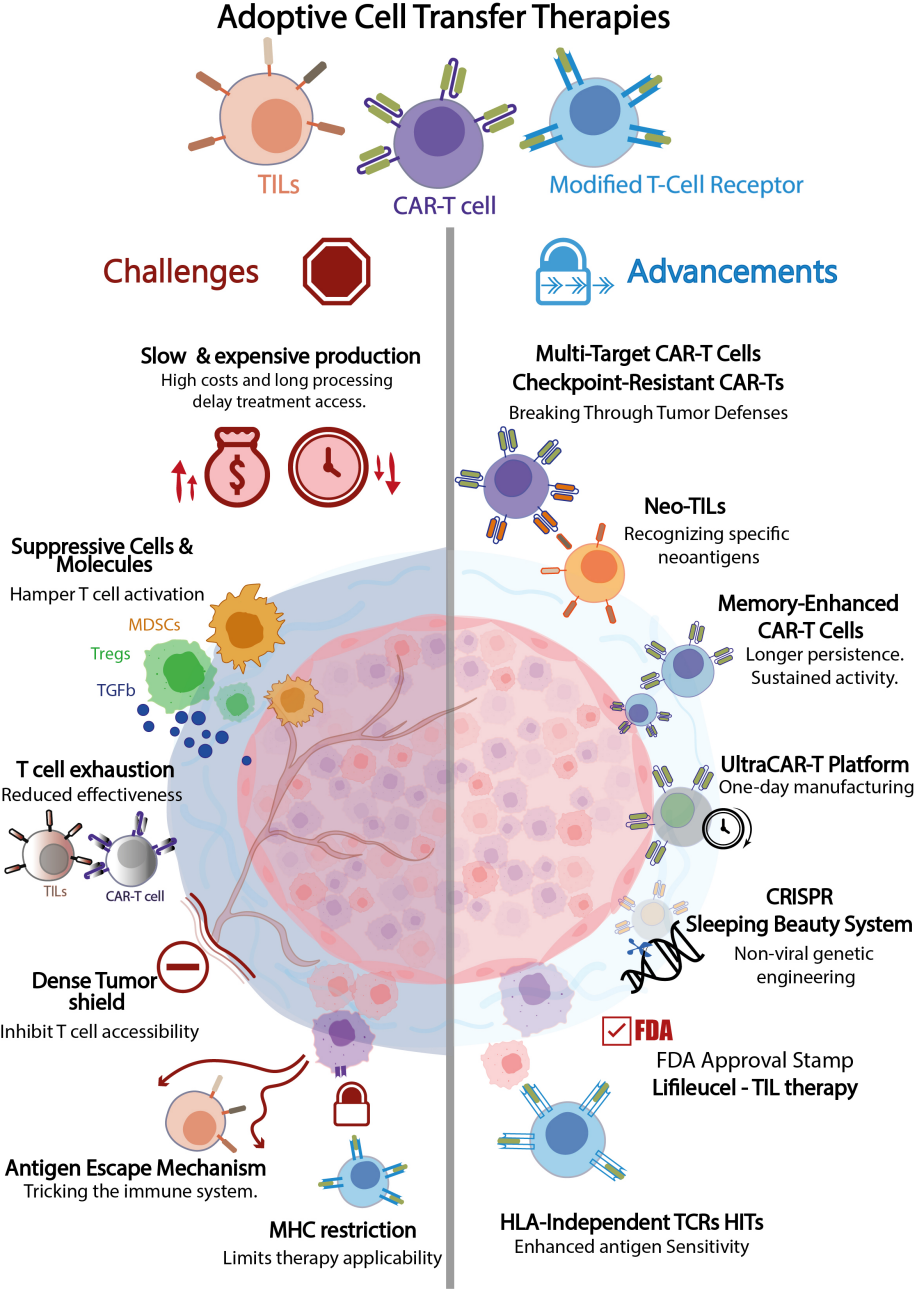
Illustration of key challenges and emerging innovations in adoptive cell transfer (ACT) therapies, emphasizing the need for continued advances in immunotherapy. The complexity of the tumor microenvironment (TME), antigen escape mechanisms, and limitations in T-cell persistence remain significant barriers to effective treatment. Recent advances, such as multi-target CAR-T cells, checkpoint-resistant CAR-Ts, and CRISPR-based genetic modifications, aim to address these challenges. Furthermore, non-viral gene transfer methods, including the Sleeping Beauty transposon System, are being explored as safer and cost-effective alternatives for T-cell engineering. Accelerated and decentralized CAR-T manufacturing platforms illustrate emerging strategies to improve accessibility. Figure created by the authors based on published literature.

Achieving these goals would expand treatment options and provide a lifeline to patients with limited therapeutic alternatives, highlighting the urgency of advancing next-generation cell therapies in cancer treatment. Given the diversity of ACT therapies in development, understanding their mechanisms, applications, and challenges is critical to maximizing their clinical impact. **Figure 1** provides a visual summary of the main barriers to ACT success, including immune suppression, antigen escape, and manufacturing constraints, as well as emerging innovations aimed at overcoming these challenges and improving therapeutic efficacy.

Additionally, as research progresses, multiple ACT platforms have evolved, each with distinct advantages and limitations. **Table 1** provides a comparative overview of key adoptive cell transfer approaches, summarizing their mechanisms of action, clinical indications, and current directions in translational and clinical development.

**Table 1:** Overview of adoptive cell transfer therapies in cancer immunotherapy.

Therapy type	Mechanism of action	Indications	Key challenges	Innovative approaches	Current research focus
CAR-T Therapy	Genetically modified T cells engineered to recognize and attack specific cancer cells.	Leukemia, lymphoma, multiple myeloma, and solid tumors (under investigation).	Immunosuppressive tumor microenvironment (TME), high costs, and complex manufacturing.	Next-generation CAR-T with CRISPR, multi-antigen CARs, and immune checkpoint inhibitors (ICIs).	Development of multi-target and armored CAR-Ts; off-the-shelf CAR-T therapies
TIL Therapy	Expansion of tumor-derived T cells ex vivo to target multiple tumor antigens.	Advanced melanoma, non-small cell lung cancer, cervical cancer and other solid tumors.	Difficulty in expanding tumor-specific T cells efficiently for all patients.	Biomarker-based patient selection, neoantigen-specific cell expansion.	Combination with ICIs, optimizing lymphodepletion, and selecting tumor-specific T cells.
TCR Therapy	Engineered T cells expressing high-affinity receptors for cancer antigens.	Solid and hematologic cancers.	Limited activation and expansion of anti-tumor T cells.	Optimized TCR receptors for improved affinity and specificity.	Development of synthetic TCR approaches and affinity-enhanced TCRs.
UltraCAR-T^®^	Rapid CAR-T manufacturing with integrated genetic enhancements for improved safety and efficacy.	Hematologic and solid tumors.	Immunosupressive TME	Manufacturing process reduced to less than 24 hours, improved persistence, and enhanced safety.	Intrinsic ICI, autoimmune indications.
Natural Killer (NK) Cell Therapy	Modified NK cells target tumors independently of specific antigens.	Solid and hematologic cancers, particularly in immunosuppressive microenvironments.	Limited persistence and cytotoxicity in harsh TMEs.	CRISPR-edited NK cells, CAR-NK development, iPSC-derived NK cells.	Development of off-the-shelf NK therapies, in combination with ICIs, and improved expansion protocols to enhance cytotoxicity in TMEs.

The following sections are organized to reflect the translational progression of adoptive cell therapies, from biological mechanisms to clinical application and implementation challenges.

## 3 Next-generation cellular therapeutics: enhancing efficacy and expanding clinical applications

### 3.1 Tumor-infiltrating lymphocyte therapy in melanoma

ACT in oncology encompasses multiple strategies designed to enhance antitumor immune responses in patients with advanced cancer. During the congress, leading experts presented recent advances in TIL-based therapies and next-generation engineered T-cell platforms, highlighting clinical outcomes, predictive biomarkers, and emerging approaches to improve efficacy, durability, and accessibility. Among these, TIL therapy, originally developed by Dr. Steven Rosenberg’s group at the National Cancer Institute in 1986, remains a foundational strategy that has evolved through decades of clinical refinement into a viable therapeutic option for patients with limited alternatives (17).

Dr. Rosenberg’s group published groundbreaking results demonstrating the effectiveness of TIL therapy in patients with metastatic melanoma. Their approach combined lymphodepletion, a single TIL infusion, and IL-2 administration, achieving an impressive overall objective response rate of 55%. Furthermore, up to 20% complete responses have been reported even in patients with substantial tumor burden, including extensive liver metastases and brain metastases, and importantly, maintained for over 10 years. These results underscore the potential of TIL therapy to achieve durable, long-term remission in some patients with advanced cancer (18, 19).

A systematic review and meta-analysis evaluating the efficacy of TIL-based therapy in melanoma patients treated across various centers revealed that adoptive cell transfer (TIL-ACT), particularly when combined with high doses (HD) of IL-2 (defined as ≥720,000 IU/kg), provides durable clinical benefits (20). Notably, most of the patients who achieved a complete response (CR) with HD-IL-2 therapy remained in remission throughout the follow-up period after their initial CR (20). These findings highlight the potential of TIL-ACT, particularly in conjunction with HD-IL-2, as a highly effective therapeutic strategy for achieving sustained remission in melanoma patients.

The approval of immune checkpoint inhibitors (ICIs) for the treatment of melanoma has significantly influenced the landscape of TIL therapy in this tumor type. A recent Phase III randomized study demonstrated superior clinical responses in patients with unresectable advanced melanoma who were treated with TILs compared to those receiving Ipilimumab. This shift points out the growing recognition of TIL therapy as a potent and effective option, particularly for patients who may not respond optimally to standard ICIs treatments (21).

In February of 2024, TIL therapy became an approved autologous cellular treatment for unresectable and metastatic melanoma, following the U.S. Food and Drug Administration (FDA) approval of a standardized TIL product, lifileucel (Amtagvi, Iovance Biotherapeutics, Inc.) (22). This accelerated approval was granted based on the results of the C-144–01 study, which evaluated patients with advanced melanoma who experienced disease progression after treatment with ICIs and targeted therapies, in cases where BRAF mutations were present. This milestone highlights the growing role of TIL therapy as a viable and promising option for patients with limited therapeutic alternatives, paving the way for expanded access to advanced melanoma treatment (23). A meta-analysis confirmed consistent clinical benefits from TIL-ACT in melanoma patients who had received prior ICIs, reinforcing its potential to improve outcomes in advanced cancers (24).

Recent clinical evidence supports the efficacy of tumor-infiltrating lymphocyte (TIL) therapy in advanced cutaneous melanoma in the current immuno-oncology era. An updated systematic review and meta-analysis including more than 600 patients reported pooled objective response rates ranging from 34% to 44% and complete response rates of approximately 10%, with no significant differences in clinical outcomes observed between patients previously treated with anti–PD-(L)1 therapy and those who were immune checkpoint inhibitor–naïve (24). In immune checkpoint inhibitor naïve metastatic non–small cell lung cancer (NSCLC), the objective response rate reached 42.1%, including a complete response rate of 10.5% (25).

Despite these encouraging clinical outcomes across melanoma and NSCLC, substantial interpatient variability in response to TIL therapy remains. This heterogeneity highlights the need to identify predictive biomarkers that can guide patient selection, optimize product characteristics, and improve clinical trial design.

### 3.2 Biomarker-guided optimization of TIL therapy

Identifying biomarkers associated with clinical response to TIL-ACT is essential for improving patient selection, optimizing treatment strategies, and designing more effective ACT-based clinical trials. During his presentation, Professor Coukos highlighted key characteristics that distinguish responder patients from non-responders. In general, patients who exhibit clinical responses show higher numbers of adoptively transferred cells. However, beyond quantity, the quality of the cells plays a crucial role. Responding patients typically harbor baseline tumors enriched with tumor-reactive TILs, which are more effectively mobilized during *in vitro* expansion. The final TIL products are enriched in tumor-specific CD8^+^ lymphocytes that effectively infiltrate tumors post-infusion. In contrast, non-responders are associated with tumors containing a low frequency of tumor-reactive resident clonotypes, with cell products largely composed of blood-borne clonotypes that infiltrated tumors post-ACT (26, 27).

Additionally, CD8^+^ TILs from responders exhibited increased cytotoxicity, exhaustion, and co-stimulatory signaling, characterized by upregulation of effector molecules (including granzyme B and perforin) and enhanced co-stimulatory pathways such as CD28-associated signaling, together with increased TCR activation programs. In parallel, myeloid populations in responder tumors demonstrated elevated type I interferon signaling and interferon-stimulated gene expression, indicating an immunologically active tumor microenvironment that supports productive T-cell–myeloid interaction networks rather than dominant immunosuppressive programs (26, 27).

### 3.3 Neoantigen-targeted TILs and the NeoScreen platform

In the final section of the talk, results related to the generation of tumor-infiltrating lymphocytes (TILs) specific to tumor neoantigens were presented. The identification of neoantigen-reactive T cells has traditionally relied on computational prediction pipelines combining whole-exome sequencing, HLA binding algorithms, and peptide–MHC multimer staining, approaches that have significantly advanced the field but remain limited by imperfect antigen prioritization and low sensitivity for detecting rare, patient-specific reactive clones (28, 29).

These conventional strategies are largely dependent on in silico predictions and do not directly assess T-cell functionality at early stages, resulting in the frequent identification of candidate neoantigens that fail to elicit productive T-cell responses. Moreover, multiple studies have highlighted that only a small fraction of predicted neoantigens are naturally processed, presented, and capable of driving antitumor immunity, underscoring the need for functionally driven screening approaches (30).

To overcome these limitations, scientists from the Ludwig Institute for Cancer Research (LICR) in Lausanne developed a novel *in vitro* TIL expansion and screening methodology termed NeoScreen. This platform leverages early functional exposure of patient-derived T cells to private sets of selected neoantigens presented by autologous, antigen-loaded B cells, enabling direct assessment of T-cell activation and reactivity at an early stage of expansion.

In contrast to peptide–MHC multimer staining or sequencing-based neoantigen prediction pipelines, which rely primarily on in silico binding affinity and often fail to capture functional T-cell reactivity (29, 30), NeoScreen was shown to enrich neoepitope-specific T cells and expand the diversity of tumor antigens identified threefold compared to conventional methods. This platform enables highly sensitive detection of rare, patient-specific neoantigens and supports the generation of expanded TILs with a superior concentration of neoepitope-specific T cells, enhancing therapeutic precision and efficacy (31, 32). Importantly, by prioritizing functional reactivity rather than solely predicted antigenicity, NeoScreen complements existing computational pipelines and addresses a key bottleneck in personalized TIL manufacturing.

### 3.4 Virus-specific and multi-antigen T-cell therapies

In addition to TIL therapy, next-generation T cells are being developed for both solid tumors and hematological malignancies, yielding promising results. Distinguished professor Catherine Bollard from Children’s National Hospital and George Washington University provided a comprehensive overview of the latest developments in this field.

Dr. Bollard discussed the use of antiviral T cells in immunosuppressed patients following allogeneic hematopoietic stem cell transplantation (HSCT). Post-transplant lymphoproliferative disease (PTLD) remains a significant complication of both hematopoietic stem cell and solid organ transplantation. A high percentage of PTLD patients are Epstein-Barr virus (EBV) positive, making EBV-targeted therapies a critical area of investigation. These next-generation T-cell therapies show great promise in addressing these challenging conditions by enhancing immune responses against malignancies while minimizing the risk of complications in immunosuppressed patients (33). Previous studies have shown that administering donor-derived T cells specifically targeting the Epstein-Barr virus (EBV) can effectively restore virus-specific immunity and control viral infections, achieving up to 90% protection *in vivo* (34). Beyond its use for HSCT complications, administering specific EBV latent membrane protein (LMP1/2) T cells to high-risk or multiple-relapse lymphoma patients as adjuvant therapy has led to durable complete responses with minimal associated toxicity in most treated patients. These findings highlight the potential of EBV-specific T-cell therapies to manage viral infections and improve outcomes for patients with high-risk lymphomas, offering a promising approach to combating aggressive malignancies (35).

Despite the successful clinical responses observed with donor-derived virus-specific T cells, the generation of a specific product for each patient is not feasible for widespread or urgent use, particularly when the donor is seronegative. To overcome this limitation, one approach is the development of banks of Human Leukocyte Antigen (HLA)-matched virus-specific T cells (VSTs) from normal seropositive individuals. These third-party EBV-directed T cells have proven to be a viable option, demonstrating a low rate of graft-versus-host disease (GVHD) and a high frequency of long-term overall response rates in patients with associated lymphomas following the transplantation of hematopoietic cells or solid organs. Notably, patients with central nervous system involvement have shown successful responses to this therapy (36). A cooperative clinical trial highlighted the feasibility and safety of administering third-party latent membrane protein-specific T cells (LMP-TCs) to pediatric solid organ transplant (SOT) recipients with PTLD treated with rituximab. Durable LMP-TC responses were observed in newly diagnosed pediatric patients with PTLD after an incomplete response to rituximab (37). Results using other viruses and pathogens as targets have been published recently (38).

Multi-target tumor-associated antigen–specific T cells are increasingly proposed as a treatment strategy for relapsed hematologic malignancies. Patients with acute myeloid leukemia (AML) who relapse after allogeneic bone marrow transplantation (BMT) have a particularly poor prognosis and limited response to conventional salvage therapies, with reported 1-year overall survival rates of approximately 20%, especially when relapse occurs within the first six months post-transplant (39).

In a recent clinical study, multi-targeted donor-derived TAA-specific T cells targeting WT1, PRAME, and survivin were administered to patients with relapsed or high-risk acute leukemia following BMT. This approach was shown to be safe and well-tolerated. Notably, patients who received TAA-T cells in a preemptive or adjuvant setting prior to overt relapse demonstrated superior clinical outcomes compared with those treated after relapse. In addition, long-term persistence of infused T-cell receptor clonotypes was observed in responding patients, supporting the durability and biological relevance of this therapeutic strategy (40).

Extending engineered T-cell therapies to solid tumors presents substantially greater challenges than hematological malignancies, primarily due to limited tumor accessibility and the presence of a hostile tumor microenvironment (41). Advanced T-cell engineering strategies, such as tumor-associated antigen cytotoxic T cells (TAA-Ts) targeting WT1, PRAME, and Survivin, aim to overcome key barriers in solid tumors, including antigen heterogeneity and immune escape. These tumor-associated antigens are intracellular proteins aberrantly overexpressed across a broad range of hematologic and solid malignancies, while exhibiting limited or tightly regulated expression in normal adult tissues. Functionally, WT1, PRAME, and Survivin play central roles in tumor cell proliferation, survival, and resistance to apoptosis, making their loss less tolerable for cancer cells and thereby reducing the likelihood of immune escape. Importantly, targeting these antigens through HLA-restricted, T-cell receptor–mediated recognition enables effective tumor killing, independent of surface antigen density, thereby partially overcoming the antigen heterogeneity and antigen-loss mechanisms that frequently limit the efficacy of CAR-T cell therapies in solid tumors (42–45). A Phase I clinical trial demonstrated the safety, feasibility, and preliminary signals of clinical activity of this approach, including disease stabilization, prolonged progression-free survival, and evidence of antigen spreading, even in the absence of lymphodepletion. These findings emphasize the potential of multi-targeted T-cell therapies as a promising option for patients with refractory or relapsed solid tumors (41).

### 3.5 Engineering resistance to the tumor microenvironment

Building on these promising results, various strategies are being explored to enhance cancer therapy outcomes. Efforts include identifying more effective targets, such as neoantigens, and utilizing tumor-specific T cells from partially HLA-matched donors, which are currently under clinical investigation. Researchers are also modifying the TME to improve T-cell infiltration and persistence, boosting adoptive cell therapy efficacy. One approach involves engineering T cells to express a dominant-negative TGF-β type 2 receptor (DN-TGF-βRII), which blocks TGF-β signaling and enhances resistance to immunosuppression. A dose-escalation study showed that TGF-resistant, EBV-specific T cells expanded and persisted safely in EBV+ Hodgkin lymphoma patients following systemic intravenous infusion in a repeated-dose schedule without lymphodepletion, achieving complete responses even in resistant cases (46). These findings underscore the potential of advanced T-cell engineering to overcome immunosuppressive barriers and improve treatment for hard-to-treat malignancies.

Based on these findings, this strategy appears safe, feasible, and capable of counteracting a significant tumor immune evasion mechanism. It could potentially be applied to other tumors, such as medulloblastoma and glioblastoma, which exploit this potent immune escape mechanism. Previous studies expanded this therapeutic approach to include cord-blood-derived NK cells as an “off-the-shelf” cell therapy, demonstrating high cytolytic activity even in the presence of TGF-β. This highlights the versatility and potential of engineered immune cells to target tumors with complex immune evasion mechanisms, offering new hope for patients with challenging malignancies (47, 48). In addition, TGFBRII DNR may rescue not only specific T cells but also CAR-T cells from immune suppression. The combination of TAA-Ts and CAR-T cells could produce a synergistic effect, as cytotoxic factors released by CAR-T cells in the TME may induce upregulation of HLA-I expression, enhancing TAA-T efficacy.

### 3.6 Next-generation CAR-T cell design and emerging indications

In solid tumors, CAR-T cell efficacy is further compromised by the immunosuppressive tumor microenvironment, antigen heterogeneity, limited intratumoral infiltration, progressive T-cell exhaustion, and the risk of on-target/off-tumor toxicity. Together, these mechanisms restrict CAR-T cell access and dampen antitumor immune activity, thereby reducing treatment efficacy. To overcome these limitations, multiple strategies are being pursued, including multi-targeted CAR-T cells, cytokine-armed fourth-generation CAR-T cells, CRISPR gene editing, and combination therapies with ICIs (anti-PD-1 and anti-CTLA-4). Ongoing clinical trials continue to explore innovative approaches to enhance the safety and efficacy of CAR-T therapy in solid tumors (16, 49).

Beyond oncology, emerging clinical evidence suggests that CAR-T cell therapies may also apply to immune-mediated diseases. Early clinical trials have demonstrated the potential of CD19-directed CAR-T cells in treating refractory autoimmune diseases, such as systemic lupus erythematosus and systemic sclerosis (50, 51). These findings highlight the growing versatility of engineered T-cell therapies and their expanding role in precision medicine​.

### 3.7 Ultra-fast and decentralized CAR-T manufacturing

Beyond biological challenges, CAR-T cell manufacturing introduces substantial logistical and scalability constraints. Accumulating clinical evidence demonstrates that prolonged vein-to-vein time adversely impacts response rates, survival outcomes, and toxicity profiles in patients with hematological malignancies treated with CAR-T cell therapies (52). The logistics of centrally manufactured products can result in the delay of treatment in these high-risk diseases and can lead to the progression of the malignancies while the patient is waiting for the product to be delivered from the central manufacturing to the treatment center (53). Additionally, FDA-approved products are manufactured by transducing the patient’s T cells with a suitable vector, followed by an extended cultivation and expansion process using cytokines. However, this prolonged *ex vivo* phase can lead to T-cell exhaustion, potentially reducing their proliferative capacity and therapeutic effectiveness after infusion (54). Therefore, methods to shorten the *ex vivo* generation of CAR-T cells are the focus of current research.

Dr. David Wald from KURE.AI presented an innovative approach to ultra-fast CAR T-cell manufacturing, achieving production in less than one day. The primary goal of this method is to preserve T-cell stemness, which enhances persistence, proliferation, cytotoxicity, and contributes to immunological memory.

This Ultra-Fast CAR (UF-CAR) manufacturing workflow involves a streamlined process of blood draw, T-cell activation, gene transfer, and formulation. Unlike conventional CAR-T cell manufacturing, UF-CARs incorporate novel domains in the scFv fragments and transmembrane regions, optimizing functionality (55). At the molecular level, the scFv domain is rationally engineered to maintain high antigen affinity and specificity while minimizing spontaneous receptor clustering and tonic signaling, which are known drivers of premature T-cell activation and exhaustion. In parallel, optimization of the transmembrane region enhances CAR stability at the cell surface and promotes more efficient coupling with intracellular signaling domains, resulting in controlled signal initiation primarily upon target engagement. Together, these structural design features are intended to support robust antigen-dependent activation while preserving a less differentiated, memory-like T-cell phenotype, an effect that may be further reinforced when combined with ultra-short ex vivo manufacturing strategies (56–58). These design and manufacturing features are intended to preserve naïve and early memory T-cell subsets and limit exhaustion, phenotypes that have been associated with improved efficacy and reduced toxicity in CD19 CAR-T therapy (54).

Dr. Wald further presented results from a Phase I clinical trial evaluating UF-KURE19 in patients with relapsed/refractory non-Hodgkin’s lymphoma (ClinicalTrials.gov identifier: NCT05400109). Patients underwent lymphodepletion with cyclophosphamide and fludarabine, followed by CAR-T cell infusion on day 0. Manufacturing was successful in all 10 patients, and the final products met all predefined release criteria. With a median follow-up of 6 months (range 1.6–14.4), the overall response rate was 80%, with all 8 responders achieving a complete metabolic response, which was maintained at the time of data cutoff (55). Importantly, the observed safety profile was favorable. CRS occurred in three patients (one grade 1 and two grade 2 cases), and one patient developed grade 3 neurotoxicity (ICANS), which resolved within one day. This favorable safety profile may be related to the delayed peak expansion of UF-KURE19 CAR-T cells, occurring at a median of approximately 21 days (range 6–30 days) (55). Additionally, circulating CAR-T cells persisted at >1% of total T cells for a median of 90 days (range 30 days to 1 year), suggesting prolonged *in vivo* activity (55).

Beyond UF-CAR approaches, a broader landscape of decentralized, non-viral, and memory-enriched CAR-T platforms, such as UltraCAR-T^®^, transposon-based systems, and genome-editing strategies, is rapidly emerging and is discussed in detail in the following section, highlighting their translational and clinical impact. However, translating these innovations into routine clinical practice remains challenging.

### 3.8 Memory-enriched CAR-T platforms, academic programs, and translational manufacturing

Despite this scientific progress, logistical and manufacturing challenges remain a major obstacle in CAR-T cell therapy. The current CAR-T production process is not only expensive, complex, and time-consuming but also difficult to scale, leading to a production rate that fails to meet the growing medical demand. This delayed manufacturing timeline often results in treatment failure, as patients with rapidly progressing diseases may experience disease worsening between cell collection and therapy administration.

To improve patient access while maintaining quality control and cost-effectiveness, extensive research is focused on addressing manufacturing scalability, high treatment costs, therapy-related toxicities, and the *in vivo* functionality of CAR-T cells. In response to these challenges, Precigen, a pioneering biopharmaceutical company, is leading innovation in next-generation gene and cell therapies targeting immuno-oncology, autoimmune disorders, and infectious diseases. Through cutting-edge technology, Precigen aims to overcome these critical barriers and advance the future of personalized cellular immunotherapy.

During the congress, Dr. Helen Sabzevari, President and CEO of Precigen, discussed the major limitations of the current autologous CAR-T cell manufacturing model and introduced UltraCAR-T^®^™, Precigen’s proprietary multigenic autologous CAR-T platform. Developed entirely in-house, UltraCAR-T™ features a decentralized manufacturing process designed for efficient technology transfer and implementation as close as possible to the patient’s clinical site. The platform utilizes a GMP-compliant, semi-closed, electroporation system, UltraPorator^®^, and for non-viral gene transfer. The UltraPorator^®^, combined with an advanced software solution, enables the cost-effective, rapid, and scalable manufacturing of personalized UltraCAR-T^®^™ therapies. Dr. Sabzevari emphasized UltraCAR-T™ as an innovative and effective solution that combines optimized manufacturing procedures with advanced genetic engineering. This approach equips CAR-T cells with additional genetic modifications, including a safety/kill switch and intrinsic checkpoint inhibition, enhancing their ability to perform multiple functions *in vivo.*

Despite the long-standing success of *ex vivo* viral transduction in achieving efficient gene transfer, safety, and *in vivo* persistence in adoptive T-cell therapy, several critical challenges continue to limit its full potential, thereby hindering the broader application of cell therapy (59). Viral vectors present significant drawbacks, including high immunogenicity, restricted cargo capacity, random genomic integration, potential insertional oncogenesis or clonal expansion, high production costs, and limited global manufacturing capacity (60). These limitations underscore the need for alternative, more efficient, and scalable gene delivery systems to advance the field of cell therapy.

Although several tens of thousands of patients globally have received CAR-T cells generated from viral vector-based delivery, in November 2023, the FDA announced safety concerns regarding secondary malignancy, highlighting 20 cases of T cell lymphoma among patients treated with CAR-T cell therapies. Still, the rate of these secondary malignancies remains low compared with other cancer treatments (61, 62). In contrast, non-viral delivery platforms are rapidly gaining popularity (63) and present a promising alternative approach, resulting in stable transgene integration and alleviating these viral-related limitations. Non-viral CAR-T cell production can be achieved through either a transposon-based system, such as Sleeping Beauty (64, 65) and PiggyBac (66), or via CRISPR/Cas, precise genome editing (67).

Compared with conventional autologous CAR-T products generated by viral transduction in centralized facilities, these non-viral platforms differ both in the underlying gene-delivery chemistry and in the operational manufacturing model. Sleeping Beauty and PiggyBac systems use plasmid DNA and a transposase rather than viral vectors, enabling lower-cost, scalable, and potentially more decentralized manufacturing. Clinically, SB-engineered CD19 CAR-T cells have shown durable engraftment, sustained *in vivo* persistence, and meaningful disease control with a favorable safety profile in heavily pretreated B-ALL and NHL patients (68). Non-viral piggyBac transposon systems provide a scalable and cost-effective alternative to viral vectors for CAR-T cell manufacturing, enabling stable CAR expression without bacterial DNA elements and facilitating clinical translation (69).

The clinical applications of non-viral delivery systems in CAR-T cell therapy are rapidly expanding and increasingly mirror those of viral vector–based approaches. Recent comprehensive reviews highlight growing clinical and translational interest in non-viral gene delivery strategies, including transposon/transposase systems and CRISPR/Cas-based genome editing, as alternatives to viral vectors for CAR-T cell engineering. These approaches aim to reduce manufacturing cost, regulatory burden, and logistical complexity while enabling more flexible and scalable CAR-T production. Although non-viral platforms currently face challenges such as lower knock-in efficiency and reduced transgene expression compared with viral systems, ongoing advances in vector design, manufacturing workflows, and quality control are steadily improving their translational feasibility across hematologic, solid, and selected non-malignant indications (70).

Despite their potentially superior biosafety profile and reduced genotoxic risk compared with viral transduction systems, non-viral methods pose unique challenges, including reduced cell recovery and lower transgene insertion efficiency. Several studies have reported substantial cell loss associated with non-viral gene delivery, which may necessitate additional ex vivo expansion to restore cell viability and could ultimately influence CAR-T cell functionality (70). Indeed, these techniques exhibit low knock-in efficiency and tend to produce lower CAR delivery and expression levels. As a result, current gene transfer methods require manual sample handling, increasing the risk of contamination, necessitating multiple manufacturing batches, and demanding extensive processing time to produce a single dose. Dr. Helen Sabzevari addressed these challenges by introducing Precigen’s UltraPorator^®^, a cutting-edge electroporation system capable of processing billions of T cells in under 12 minutes. This innovation enables a semi-closed, overnight manufacturing process, followed by standardized quality control procedures, allowing for patient administration just one day post-gene transfer within the same medical center. This streamlined approach aligns with the clinical definition of “vein-to-vein” time, significantly improving efficiency and accessibility. To fully harness the potential of CAR-T cell functionality and biology *in vivo*, multiplexed and precise genome engineering technologies are emerging as key enablers for designing CAR-T cells with customized genomes. These advances ensure high and stable CAR expression, targeted functionality, long-term persistence, and robust *in vivo* activity. Notably, non-viral Sleeping Beauty vectors offer a major advantage over viral-based methods, as they impose no strict limitations on transgene size (71), providing greater flexibility for genetic modifications.

Dr. Helen Sabzevari introduced the UltraVector^®^ DNA construction, a large multigenic payload, maintained indefinitely as plasmid DNA, simple, less costly to manufacture with high integration efficiency used in the UltraCAR-T™^®^ platform. As a result, the UltraCAR-T™^®^ cell product simultaneously expresses an antigen-specific CAR tailored and fine-tuned to bind target cells and to meet the unique requirements of each clinical application, features a safety/kill switch mechanism for controlled elimination of CAR-T cells if needed for safety profile, and incorporates *in vivo* function such as the expression of membrane-bound interleukin-15 (mbIL15). IL-15 is a critical modulator cytokine involved in maintaining the naïve, effector, and memory T cells, but it also plays a predominant role in the reactivation of memory T cells and their survival to enhance anti-tumor response (72).

The preservation of an immature phenotype in CAR-T cells is associated with a longer cell lifespan and is critical for achieving extended survival times in patients. Indeed, Dr. Helen Sabzevari pointed out that the expression of mbIL15 on UltraCAR-T™^®^ cell product showed no need for *ex vivo* expansion of T cells before cell administration as well an enhancement of *in vivo* expansion in the presence of tumor antigens combined with longer persistence, less exhaustion, and enduring anti-tumor response. This is in line with the addition of IL-7/IL-15 to the cultured CAR-T cells in the automated CliniMACS Prodigy^®^ manufacturing platform, to result in a quiescent cellular profile despite extensive activation and expansion (73). Noteworthy, guided by the UAE’s leadership, ADSCC is advancing cancer and autoimmune treatments using the automated CliniMACS Prodigy^®^ platform in manufacturing CAR-T cells and “vein to vein” treating almost 20 patients across the region to date (74).

In addition, Dr. Helen Sabzevari presented promising data on Precigen’s UltraCAR-T™^®^ investigational cell products, PRGN-3006, -3007, -3008, and -3005, and their related clinical programs, showing the potential of Precigen’s cutting-edge approaches to cell and gene therapy in the treatment of hematological and solid cancers and autoimmune diseases. She first presented PRGN-3006 UltraCAR-T™^®^ cells, utilizing Precigen’s UltraVector^®^ platform, tailored to express CD33-CAR with high specificity to kill CD33+ myeloid leukemia cells and leukemic stem cells. Interestingly, the FDA has granted “Fast Track” and orphan drug designation to PRGN-3006 UltraCAR-T™^®^, as a candidate in a human Phase 1 clinical trial (NCT03927261) for the treatment of patients with relapsed or refractory (r/r) AML and higher-risk myelodysplastic syndrome (MDS) diseases (75).

Despite the significant achievements of CAR cell therapy, its effectiveness in solid tumors remains limited due to the challenges posed by TME. These include physical obstacles like irregular vasculature and a fibrogenic extracellular matrix; immunosuppressive factors such as cytokines, myeloid-derived suppressor cells, tumor-associated macrophages, regulatory T cells; and metabolic competition in a hypoxic, nutrient-poor TME that supports tumor growth while impairing CAR T-cell function (76). To overcome these roadblocks, Precigen has enhanced the UltraCAR-T^®^ platform with innovative strategies to counteract the inhibitory TME without requiring complex or costly gene editing. PRGN-3007 is engineered using a single multicistronic transposon plasmid to co-express Receptor Tyrosine Kinase-like Orphan Receptor 1 (ROR1)-CAR, an intrinsic PD-1 blockade, mbIL15, and a kill switch, eliminating the need for systemic checkpoint inhibitors. Given ROR1 overexpression in CLL, MCL, ALL, and TNBC, a Phase 1/1b open-label trial is designed to evaluate PRGN-3007 in patients with advanced ROR1+ hematologic (Arm 1) and solid tumors (Arm 2). The trial follows a 3 + 3 dose escalation phase, leading to dose expansion at the maximum tolerated dose (MTD), with parallel enrollment in both arms. This investigator-led study is conducted in collaboration with the H. Lee Moffitt Cancer Center & Research Institute, Tampa, Florida, USA.

PRGN-3007 and PRGN-3008 are similarly engineered, with the latter co-expressing CD19-CAR and the intrinsic PD-1 blockade to harbor the potential to be the best-in-class treatment for B-cell malignancies and autoimmune indications. Presented preclinical data showed that *in vivo* tumor models, PRGN-3008 enhanced expansion and persistence, produced antitumor efficacy with complete tumor clearance, and demonstrated significantly longer survival compared to conventional CD19 CAR-T cells. In a simulation of tumor relapses in the *in vivo* model, PRGN-3008 demonstrated persistence and long-term antitumor immunity, extending overall survival without additional PRGN-3008 treatment. Furthermore, in a humanized mouse model of lupus nephritis, additional preclinical data for PRGN-3008 showed complete clearance of B-cells as well as a decrease in antibodies to double-stranded DNA, a specific marker of lupus. In ovarian cancer, some clinical studies have recently started to explore the feasibility, safety, and anti-tumor activity of CAR cell technology using T and NK cells targeting tumor-associated antigens such as MSLN, MUC16, TROP2, TAG72, and MICA (77). PRGN-3005, an UltraCAR-T cell product manufactured to express a CAR targeting unshed Mucin 16 (MUC16), a cell‐surface antigen overexpressed in several epithelial cancers, including most ovarian cancer, membrane-bound IL-15 (mbIL15), and the kill switch, has been evaluated in 25 relapsed or refractory (r/r) ovarian cancer patients (pts) that have limited treatment options. The investigational product has been well tolerated with minimal toxicity and demonstrated a dose-dependent expansion and persistence in blood with or without lymphodepletion for up to 9 months post-infusion. Encouraging disease control rates and a reduction in overall tumor burden have been observed in heavily pretreated ovarian cancer patients treated with lymphodepletion. The Phase 1b clinical study (NCT03907527) is designed to evaluate ovarian cancer patients with high-dose therapy following a lymphodepletion regimen, with an option to receive a second dose (78). Dr. Helen Sabzevari concluded her talk by sharing Precigen’s vision, which aims to amplify and deliver an off-the-shelf library of UltraVector^®^ sequences tailored to a patient’s tumor, enabling the production of personalized autologous UltraCAR-T^®^ therapies with multiple genetic edits or transgene insertions. This approach leverages overnight manufacturing at the patient’s medical center, streamlining treatment delivery and enhancing accessibility.

Beyond optimizing manufacturing efficiency, enhancing CAR-T cell biology is equally critical for improving therapeutic outcomes. Research indicates that less differentiated T-cell subsets are strongly associated with greater therapeutic efficacy. Among these, central memory T cells (T_CM_) and memory stem T cells (T_SCM_) exhibit superior proliferative capacity, persistence, and antitumor effects compared to effector memory T cells (T_EM_) and effector T cells (T_EF_). Notably, T_SCM_ cells, with their self-renewal ability and capacity to generate both memory and effector T-cell progeny, represent a promising target for enhancing CAR-T cell durability and potency. Enriching T_SCM_ and T_CM_ subsets in the final CAR T-cell product has been shown to enhance clinical efficacy (79, 80). Advances in culture conditions, including the use of IL-7, IL-15, and IL-21, combined with strategies such as CD62L^+^ selection, have demonstrated promise in promoting T_SCM_ generation. These novel approaches have the potential to maximize the therapeutic impact of CAR-T cell therapy by improving persistence, proliferation, and antitumor efficacy (80, 81).

In this context, Dr. Briones introduced an academic CAR-T cell program for hematologic malignancies within the Hematology Service of Hospital Sant Pau, Barcelona. As part of this initiative, Dr. Briones presented the development of a CAR-T cell platform enriched in memory T cells (T_SCM_ and T_CM_) subsets known for their superior proliferative capacity, persistence, and antitumor activity. This strategy aims to enhance therapeutic efficacy, addressing key challenges in CAR-T therapy durability and long-term clinical response. This second-generation CAR is designed to specifically target CD30, a molecule highly expressed in Hodgkin lymphoma cells, while being largely absent in healthy tissues. The CAR construct incorporates 4-1BB co-stimulation and utilizes an antibody designed to recognize a membrane-proximal epitope of the non-soluble portion of CD30, thereby avoiding inhibition by soluble CD30. Additionally, design features that promote higher CAR expression levels have been shown to enhance antitumor activity without inducing functional exhaustion, supporting the relevance of optimizing CAR architecture and T-cell quality to improve persistence and therapeutic efficacy (82).

Preclinical studies demonstrated that a single dose of CD30-CAR T_SCM_-enriched cells effectively eradicated established tumors and exhibited long-term persistence *in vivo*. This durability was evidenced by the cells’ ability to eliminate tumors upon rechallenge without requiring additional CAR T-cell administration. Moreover, a substantial presence of CAR T cells was observed in lymphoid organs, with over 50% of these cells identified as T_SCM_ or T_CM_ cells, further supporting their potential for sustained antitumor immunity (83).

During his presentation, Dr. Briones highlighted the progress of the academic platform for CD30-CAR T_SCM_-enriched cells, which has successfully advanced to Phase I/IIa clinical trials. This approach has shown promising outcomes in patients with advanced Hodgkin lymphoma, with 50% achieving a complete response following CD30-CAR T cell treatment (84). Building on this success, Dr. Briones expanded the concept to develop a second academic CAR-T cell platform targeting CD19. This CD19-CAR T cell product is manufactured by isolating less differentiated CD62L+ T cells, aiming to generate a final product highly enriched in T_SCM_ and T_CM_ subsets, which may enhance clinical efficacy and treatment durability.

As these academic CAR-T programs progress, broader research efforts continue to push the boundaries of therapeutic innovation. Next-generation CAR T cells are being engineered to secrete cytokines, thereby enhancing T-cell antitumor activity while modulating the TME to improve treatment response. Additionally, novel CAR T cells with switch receptors are being designed to block inhibitory tumor signals, effectively overcoming key mechanisms of immune evasion (85).

These breakthroughs highlight the complexity of CAR-T therapy and underscore the critical need for continuous refinement in areas such as cell selection, genetic engineering strategies, and tumor-targeting mechanisms. By advancing these approaches, researchers aim to maximize clinical outcomes and expand the therapeutic potential of CAR-T cells across a broader range of malignancies, ultimately bringing more effective and durable treatments to patients in need.

## 4 Synthetic immunity: unlocking mechanisms to transform T-cell therapeutics

### 4.1 Antigen escape, trogocytosis, and dual-targeting CAR designs

As researchers continue to refine CAR-T therapy, the concept of synthetic immunity is emerging as a transformative force in T-cell therapeutics, aiming to overcome key challenges in cancer treatment. One of the most significant hurdles in this regard is antigen escape, a mechanism that enables tumors to evade immune detection and resist therapy. During the workshop, Mohamad Hamieh from Weill Cornell Medicine in NY provided an in-depth analysis of this phenomenon, emphasizing its profound impact on tumors with low or absent antigens, which pose a major challenge to CAR-T cell therapy. He specifically highlighted the role of trogocytosis, a process in which T cells acquire target antigens from tumor cells, inadvertently facilitating immune evasion. This antigen transfer reduces the density of target antigens on tumor cells, leading to T-cell exhaustion and fratricide, ultimately diminishing CAR-T cell effectiveness and contributing to treatment resistance and disease relapse (86).

To counteract antigen-low relapse and prevent tumors from evading immune surveillance, dual-targeting strategies have emerged as a promising approach in CAR-T therapy. These strategies aim to regulate the antigen density threshold required for CAR-T cell activation by rationally fine-tuning CAR design and signaling. A key advancement in this area is the development of dual-CAR T cells or tandem-CARs, which are engineered to simultaneously target two different antigens, thereby reducing the likelihood of tumor escape (86). These approaches are already being explored in clinical trials for B-cell malignancies and multiple myeloma (MM). Early results suggest that dual targeting is both feasible and safe; however, further research is needed to determine whether this strategy provides superior efficacy compared to traditional single-target CAR-T cells. As the field progresses, optimizing dual-targeting mechanisms may be the key to overcoming immune evasion and enhancing the durability of CAR-T therapies (87).

### 4.2 HLA-independent T-cell receptors, synthetic co-stimulation and combinatorial targeting

Building on these innovations, Dr. Hamieh described another promising advancement in CAR-T therapy: the use of HLA-independent T-cell receptors (HITs), designed to enhance antigen recognition, particularly for low-abundance targets. Unlike conventional CARs, HIT receptors are engineered to replace or functionally bypass the endogenous TCR with a high-affinity synthetic receptor that significantly improves sensitivity to tumor-associated antigens. In preclinical studies, T cells engineered with HIT receptors exhibited approximately 10-fold greater antigen sensitivity compared to 28ζ CAR T cells. Remarkably, in a mouse xenograft model, these HIT-engineered T cells successfully controlled leukemia with as few as 200 CD19 molecules per cell, demonstrating their potential to overcome the limitations of antigen density. This enhanced sensitivity makes HIT receptors particularly valuable for targeting tumors characterized by low antigen expression (88).

These advancements highlight the potential of combinatorial targeting strategies, tailored to antigen density, to effectively counteract tumor escape mechanisms, including those driven by low-antigen expression. While synthetic immunity continues to revolutionize cancer therapy, several challenges remain. Recent breakthroughs in CAR-T cell engineering, HIT receptors, and synthetic dual-costimulatory modules such as CD80–4-1BB fusion proteins and switch receptors like PD1–CD28, are enhancing the functional persistence of T-cell-based therapies, paving the way for more effective and durable treatments. By integrating multiple targeting strategies, researchers aim to improve therapeutic efficacy and broaden the application of engineered T-cell therapies beyond hematologic malignancies to solid tumors. These innovations mark a significant step forward in the pursuit of next-generation immunotherapies capable of overcoming the inherent challenges of tumor heterogeneity and immune evasion (89).

The future of synthetic immunity will depend on the coordinated integration of these technologies into clinically scalable platforms, ushering in a new era of cancer treatment. By leveraging next-generation CAR-T engineering, HIT receptors, and combinatorial targeting strategies, researchers are driving the development of personalized, precise, and durable therapies capable of overcoming immune evasion and tumor heterogeneity. As these innovations progress, synthetic immunity is poised to transform cancer care, extending its impact beyond hematologic malignancies to solid tumors, ultimately offering new hope to patients with limited treatment options.

## 5 Breaking barriers in CAR-T therapy: advancements in manufacturing, cost reduction, and global accessibility

### 5.1 Barriers to access and affordability in current CAR-T paradigms

Despite the groundbreaking potential of CAR-T therapy, a critical challenge remains to ensure equitable access and affordability, particularly for pediatric patients and individuals with rare or aggressive cancers. The high cost of manufacturing, complex regulatory hurdles, and limited commercial viability within traditional biopharmaceutical frameworks have restricted access to these life-saving therapies.

To address these challenges, scientists and healthcare advocates are calling for innovative models that streamline clinical trial registration, support biological license applications, and facilitate the commercialization of approved cell therapies for children. One transformative approach involves collaborative biotech models that integrate academic medical centers and research institutes. By leveraging academic manufacturing capabilities, this model seeks to reduce production costs, expedite regulatory approvals, and accelerate the delivery of CAR-T therapies to patients who need them most.

### 5.2 Regulatory progress and clinical expansion

At the same time, significant progress continues to be made in CAR-T therapy research and development. Currently, six CAR-T therapies have received FDA approval for the treatment of various hematologic malignancies (**Table 2**). Meanwhile, numerous other CAR-T therapies are undergoing clinical evaluation, reflecting ongoing efforts to expand therapeutic applications, enhance efficacy, and improve patient safety (90, 91).

**Table 2:** FDA-approved CAR-T cell therapies.

Product name	Year of approval	Indication
Kymriah (Tisagenlecleucel)	2017	Pediatric and young adult patients (≤ 25 years old) with R/R B-cell ALL.
Yescarta (Axicabtagene ciloleucel)	2017	Adult patients with R/R large B-cell lymphoma after two or more lines of systemic therapy.
Kymriah (Tisagenlecleucel)	2018	Adult patients with R/R large B-cell lymphoma after 2 or more lines of systemic therapy.
Tecartus (Brexucabtagene autoleucel)	2020	Adult patients with R/R ALL.
Breyanzi (Lisocabtagene maraleucel)	2021	Adult patients with R/R large B-cell lymphoma after 2 or more lines of systemic therapy.
Yescarta (Axicabtagene ciloleucel)	2021	Adult patients with R/R follicular lymphoma after 2 or more lines of systemic therapy.
Abecma (Idecabtagene vicleucel)	2021	Adult patients with R/R MM after 4 or more lines before therapy.
Carvykti (Ciltacabtagene autoleucel)	2022	Adult patients with R/R MM

ALL, acute lymphoblastic leukemia; B-cell, B lymphocyte; CAR-T, chimeric antigen receptor T-cell; FDA, Food and Drug Administration; MM, multiple myeloma; R/R, relapsed/refractory.

These developments underpin a commitment to refining CAR-T technology, ensuring that it not only achieves optimal clinical outcomes but also becomes more accessible to diverse patient populations. As research continues, next-generation CAR-T therapies, including dual-targeting approaches, synthetic immunity, and improved T-cell engineering, hold the potential to broaden the reach of cellular immunotherapies beyond hematologic cancers to solid tumors and other challenging malignancies.

### 5.3 Market growth and global demand for CAR-T therapy

The growing significance of CAR-T therapy is further reflected in its rising global demand. According to the American Society of Gene & Cell Therapy, CAR-T cell treatment is the leading technology in the pipeline of genetically modified cell therapies, accounting for 49% of all developments. Of these, an overwhelming 96% are focused on cancer indications, while the remaining therapies explore applications for HIV/AIDS and autoimmune diseases. This surge in research and development is mirrored by rapid market expansion. In 2023, the CAR-T cell therapy market was valued at USD 3.52 billion, with projections indicating a compound annual growth rate (CAGR) of 34% from 2024 to 2030. By 2030, the market is expected to reach approximately USD 27.31 billion, fueled by technological advancements, increasing regulatory approvals, and broader clinical applications. These trends underscore CAR-T therapy’s growing impact in modern medicine, highlighting its potential to transform oncology and expand into non-cancer indications. As research continues to refine next-generation CAR-T therapies, the convergence of scientific progress, regulatory support, and market growth is set to accelerate global adoption, accessibility, and therapeutic innovation (92, 93).

### 5.4 Disparities in real-world access to CAR-T therapy

Despite its transformative potential, access to CAR-T therapy remains suboptimal, raising concerns about equitable treatment availability. In the United States, CAR-T therapy is primarily offered at around 200 academic medical centers, representing only 3-5% of healthcare facilities. As a result, many oncologists do not refer to or administer CAR-T therapy, creating a significant gap between eligible patients and those who receive treatment. For instance, in the U.S., only 15-20% of Diffuse Large B-cell Lymphoma (DLBCL) cases are treated with CAR-T cell infusion, despite its potentially curative impact. This disparity means that patients continue to die from cancers that are both treatable and potentially curable through cell therapies (94).

In addition to institutional limitations, sociogeographical factors play a major role in determining real-world access to CAR-T therapy. Observational analyses have shown that many patients who ultimately receive CAR-T treatment reside in proximity to accredited treatment centers, whereas patients living farther away are less likely to receive therapy. Travel distance, caregiver availability, prolonged post-infusion monitoring requirements, and the need for temporary relocation near specialized centers represent significant barriers for patients from rural or remote areas (95).

Beyond geography, racial and socioeconomic disparities have also been reported in real-world CAR-T utilization. Patients from racial and ethnic minority groups and those with lower socioeconomic status are underrepresented among CAR-T recipients, despite comparable disease incidence and eligibility. Contributing factors include differences in referral patterns, insurance coverage, clinical trial participation, and the ability to comply with intensive follow-up and supportive care requirements (95).

Globally, access to CAR-T therapy remains highly heterogeneous. In South America, availability is largely confined to academic initiatives or compassionate-use programs, with limited local manufacturing capacity, fragmented regulatory frameworks, and restricted reimbursement pathways constraining broader implementation (96). Recent regional analyses emphasize the need for domestic manufacturing platforms, regulatory harmonization, and public–academic partnerships to enable sustainable and equitable access. In Asia, access varies widely across countries, with regions such as China and Japan advancing domestically developed CAR-T products and regulatory pathways, while many other areas continue to face infrastructural, reimbursement, and workforce-related barriers (97). In Europe, although CAR-T therapies are approved and incorporated into clinical guidelines, access remains uneven across countries, reflecting differences in national reimbursement policies, center accreditation requirements, and the concentration of treatment delivery within EBMT/JACIE-accredited cellular therapy programs, which may contribute to geographic disparities in patient access (98).

### 5.5 Economic burden and treatment-associated costs

Ensuring equitable access to CAR-T therapy remains a critical challenge, as financial, logistical, and institutional barriers limit its availability to eligible patients. The high cost of a single infusion of an FDA-approved CAR-T therapy ranges from $373,000 to $475,000, making affordability a significant barrier for both patients and healthcare systems. CAR-T production involves multiple intricate steps, from cell collection and genetic modification in centralized facilities to reinfusion, a process that can take up to six weeks, which is often too long for patients with aggressive diseases. Addressing these challenges requires innovative solutions to streamline production, reduce costs, and expand healthcare infrastructure, bridging the gap between scientific advancements and real-world patient access (94, 99).

Beyond the high upfront cost of CAR-T therapy, patients may face additional financial burdens associated with pre-treatment procedures and post-infusion care. The cost of leukapheresis (T-cell collection) and lymphodepletion therapy (conditioning chemotherapy) can add to the overall expense. Moreover, post-treatment complications often require intensive medical support, further increasing costs. Two of the most common adverse effects following CAR-T therapy are CRS and ICANS. These conditions can necessitate hospitalization, intensive care, and interventions such as IL-6 receptor-targeting monoclonal antibodies (e.g., tocilizumab) to manage severe inflammatory responses. Additionally, many patients require long-term immunosuppressive and supportive care due to B-cell aplasia and hypogammaglobulinemia, increasing their risk of infections. Consequently, lifelong medications or regular immunoglobulin replacement therapy may be needed, compounding the financial and logistical burden of CAR-T treatment (100, 101).

In addition to direct treatment and toxicity-associated costs, regulatory requirements play a critical role in shaping the real-world economic and logistical burden of CAR-T therapy. In the United States, CAR-T products are subject to Risk Evaluation and Mitigation Strategies (REMS), which are drug-specific safety programs required by the U.S. Food and Drug Administration to ensure that the clinical benefits of therapies with serious risks outweigh their potential harms (102).

For CAR-T therapies, REMS programs primarily focus on standardized training of treatment centers, early recognition of CRS and ICANS, and structured post-infusion monitoring during periods of highest risk. Importantly, as clinical experience has expanded and consensus toxicity grading systems have been established, REMS implementation has evolved toward more risk-adapted and flexible monitoring approaches, allowing selected patients to transition earlier to outpatient management when clinically appropriate (15).

This evolution in regulatory oversight has meaningful implications for accessibility and financial toxicity, as reduced dependence on prolonged inpatient monitoring and temporary relocation near certified centers may lower indirect costs related to hospitalization, caregiver burden, and travel, while maintaining patient safety through standardized REMS-compliant practices.

### 5.6 Decentralized manufacturing and future access models

During the ADSCC Congress, Dr. Daniel Couriel (Huntsman Cancer Institute, University of Utah) framed a central strategic question for the field: how can access to cell therapies be expanded while maintaining safety, efficacy, and long-term sustainability? He emphasized that high costs remain one of the most significant barriers to the broader adoption of CAR-T therapies.

In his discussion, Dr. Couriel highlighted two major areas requiring optimization: (i) infrastructure and service delivery models, and (ii) the CAR-T product itself. The current manufacturing and distribution model is highly centralized, leading to logistical bottlenecks and high operational costs, limiting access for many patients. In addition, innovative technologies and streamlined production workflows are required to enable simpler, faster, more cost-effective, and scalable manufacturing, while preserving critical product attributes such as T-cell stemness, long-term persistence, robust antitumor efficacy, and an acceptable safety profile.

One promising approach to overcoming these barriers is the decentralized production of CAR-T cells, which could reduce costs and promote more equitable access by allowing treatment centers to manufacture CAR-T cells on-site rather than relying on centralized biopharmaceutical facilities. However, implementing decentralized manufacturing requires significant investment and intensive training for both CAR-T cell production personnel and quality assurance teams, making widespread adoption challenging.

Beyond technological and logistical improvements, regulatory support plays a critical enabling role. Simplifying regulatory pathways and promoting harmonized approval processes could accelerate the availability of CAR-T therapies in more healthcare settings worldwide.

## 6 Outlook

Despite these challenges, the outlook remains optimistic. Cross-sector collaboration among researchers, clinicians, regulatory agencies, and industry partners will be essential to overcome existing barriers. Through innovative solutions and coordinated efforts, the vision of expanding access to ACT can become a reality, ensuring that this transformative therapy reaches more patients who need it most.

## 7 Conclusions

The Second Bone Marrow Transplant and Cellular Therapy Congress underscored the rapid maturation of adoptive cell therapies, highlighting major advances in CAR T-cell therapy, TIL-based treatments, and next-generation T-cell engineering, reinforcing their expanding role as a cornerstone of modern oncology. While CAR T-cell therapy has transformed the treatment landscape of hematologic malignancies, challenges persist in solid tumors due to antigen heterogeneity, immune evasion, and the suppressive tumor microenvironment. However, emerging innovations, including memory-enriched CAR T cells, dual-targeting and synthetic immunity strategies, and neoantigen-specific TIL therapies alone or in combination with immune checkpoint inhibitors, are beginning to address these barriers and broaden therapeutic applicability.

Despite these scientific breakthroughs, the congress clearly highlighted that clinical impact remains limited by high manufacturing costs, complex logistics, and uneven global access. The need for innovative biotech and translational models, including academic manufacturing frameworks and decentralized CAR T-cell production, was emphasized as a critical pathway to reduce costs, enhance scalability, and improve accessibility, particularly for pediatric patients and underserved populations.

Looking ahead, sustained collaboration among academic institutions, industry partners, regulatory bodies, and healthcare systems will be essential to bridge innovation and implementation. By aligning advances in cell engineering with optimized regulatory pathways, manufacturing innovation, and access-oriented delivery models, the full potential of cellular immunotherapy can be realized, ensuring that life-saving cellular therapies reach those who need them most worldwide.

The future impact of adoptive cell therapies will depend not only on biological innovation but also on the successful integration of manufacturing decentralization, regulatory adaptation, and equitable access models. As highlighted throughout this review, next-generation platforms that unify these dimensions are poised to define the next era of cellular immunotherapy.
